# Development of a Machine Learning-Based Predictive Model for Arteriovenous Fistula Occlusion After Surgery: A Retrospective Cohort Study from 2015 to 2025

**DOI:** 10.3390/medicina61122150

**Published:** 2025-12-02

**Authors:** Jae Hoon Lee, Sang Gyu Kwak

**Affiliations:** 1 Division of Vascular and Endovascular Surgery, Department of Surgery, School of Medicine, Daegu Catholic University, Duryugongwon-Ro 17-Gil 33, Nam-Gu, Daegu 42472, Republic of Korea; vsljh@cu.ac.kr; 2 Department of Medical Statistics, School of Medicine, Daegu Catholic University, Duryugongwon-Ro 17-Gil 33, Nam-Gu, Daegu 42472, Republic of Korea

**Keywords:** arteriovenous fistula, hemodialysis, machine learning, vascular access, occlusion

## Abstract

*Background and Objectives*: Arteriovenous fistula (AVF) occlusion remains a major cause of vascular access failure in hemodialysis patients. Early identification of high-risk patients may help prevent complications and improve outcomes. *Materials and Methods*: This retrospective cohort study included 1498 adult patients who underwent AVF creation between 2015 and 2025 at Daegu Catholic University Medical Center. Clinical, surgical, and laboratory variables were used to develop machine learning (ML) models for predicting AVF occlusion. Five algorithms—LightGBM, CatBoost, XGBoost, Random Forest, and Logistic Regression—were trained and evaluated using stratified five-fold cross-validation. Model performance was assessed using area under the receiver operating characteristic curve (AUC), accuracy, sensitivity, specificity, and calibration. SHAP (Shapley Additive Explanations) analysis was used to interpret variable importance. *Results*: Among the 1498 patients, 381 (25.4%) experienced AVF occlusion. LightGBM achieved the best performance (AUC = 0.887, accuracy = 0.858, specificity = 0.950), followed by CatBoost (AUC = 0.882) and XGBoost (AUC = 0.879). Calibration analysis demonstrated strong agreement between predicted and observed outcomes. SHAP analysis identified ferritin, hemoglobin, neutrophil percentage, and C-reactive protein as the most influential predictors, highlighting the role of inflammation and hematologic status in AVF failure. *Conclusions*: Gradient boosting-based ML models, particularly LightGBM and CatBoost, accurately predict AVF occlusion using routine clinical data. Explainable AI methods enhance interpretability, enabling early identification of high-risk patients and supporting precision vascular access management in hemodialysis care.

## 1. Introduction

Arteriovenous fistula (AVF) is widely regarded as the optimal vascular access for patients undergoing maintenance hemodialysis due to its superior long-term patency and lower complication rates compared to arteriovenous grafts or central venous catheters. Despite its advantages, AVF occlusion remains a significant clinical problem that impairs dialysis efficiency and leads to repeated interventions such as angioplasty or revision surgery, thereby increasing patient morbidity and healthcare burden [1,2]. The occurrence of AVF occlusion is influenced by multiple factors including patient demographics (age, sex), comorbidities (diabetes, hypertension), vascular anatomy, and surgical techniques [3].

Recent analyses (2022–2024) have also demonstrated evolving patterns in vascular access failure, highlighting the ongoing clinical relevance of AVF dysfunction in modern hemodialysis practice [4,5].

Previous studies have primarily investigated risk factors for AVF occlusion using traditional statistical methods such as univariate and multivariate Logistic Regression analyses [6,7,8,9]. Jin et al. [7] developed a risk prediction model for autogenous AVF thrombosis in maintenance hemodialysis patients; however, their model was limited by the relatively small number of variables and dataset size, restricting its generalizability.

Hu et al. [8] compared post-thrombotic endovascular interventions with pre-emptive angioplasty and highlighted the procedural and anatomical factors associated with AVF dysfunction, and Li et al. [9] demonstrated that puncture-related thrombosis risk can be evaluated using structured risk assessment models. Although informative, these studies did not fully capture the complex interactions among clinical variables or account for nonlinear relationships, underscoring the need for more sophisticated machine learning approaches.

Traditional regression-based models are constrained by several methodological limitations: (1) they rely on linearity assumptions that may not adequately reflect nonlinear vascular and inflammatory processes; (2) they require manual specification of interaction terms, limiting the ability to capture higher-order interactions among clinical, anatomical, and laboratory variables; and (3) they are highly susceptible to multicollinearity, which is common in hemodialysis populations and can destabilize coefficient estimates. These limitations can reduce predictive accuracy when applied to heterogeneous real-world patient data. Such constraints underscore the need for more flexible modeling approaches capable of capturing the complex relationships inherent to AVF.

Recent advances in medical big data and machine learning (ML) provide opportunities to overcome these limitations by integrating numerous clinical, laboratory, and procedural variables into predictive models with enhanced accuracy [10]. ML algorithms such as XGBoost, LightGBM, Random Forest, and Logistic Regression have demonstrated superior performance across diverse clinical prediction tasks by capturing complex patterns and interactions in data [11,12]. Furthermore, explainable AI techniques like SHAP (Shapley Additive Explanations) facilitate model interpretability, enabling clinicians to understand variable importance and effect directionality, thus improving clinical applicability.

Several recent studies have demonstrated that machine learning models outperform classical regression approaches in predicting AVF maturation, patency, or thrombosis risk, further supporting the need for ML-based prediction frameworks [13,14].

Accordingly, this retrospective cohort study aims to develop and validate ML-based predictive models for postoperative AVF occlusion using comprehensive clinical data from adult patients who underwent AVF surgery at Daegu Catholic University Medical Center between 2015 and 2025. By leveraging a large-scale dataset encompassing patient demographics, comorbidities, surgical variables, and laboratory results, we seek to construct robust predictive models and identify key risk factors. The ultimate goal is to provide a reliable tool for the early identification of high-risk patients to support timely preventive interventions and personalized management strategies, thereby improving patient outcomes and healthcare resource utilization.

## 2. Methods

The study protocol was approved by the Institutional Review Board (IRB) of Daegu Catholic University Hospital (DCUMC 2025-11-012). Data from 1 January 2015 to 30 September 2024 were retrospectively collected and analyzed. As a retrospective study using de-identified medical records, individual patient consent was waived. All data were anonymized and securely stored in compliance with relevant privacy regulations.

### 2.1. Study Design and Population

This retrospective cohort study included adult patients (≥18 years) who underwent arteriovenous fistula (AVF) surgery for hemodialysis at Daegu Catholic University Medical Center from 1 January 2015 to 30 September 2025. A total of 1660 patients were initially identified. Of these, 162 patients were excluded based on the following predefined criteria: 38 patients died or were lost to follow-up within 1 month after surgery, 74 patients had incomplete or missing essential clinical or laboratory records required for model development, 28 patients underwent AVF revision or secondary procedures rather than primary AVF creation, and 22 patients met other exclusion criteria (e.g., non-adult age, catheter-only access, or inconsistent documentation). After applying these criteria, 1498 patients were included in the final analytic cohort. A detailed flow diagram outlining the patient selection process was constructed for clarity.

### 2.2. Data Collection and Preprocessing

Clinical data, including demographic characteristics, comorbidities, laboratory results, vascular ultrasound findings, and dialysis history, were extracted from electronic medical records. Variables collected comprised age, sex, body mass index (BMI), presence of hyperlipidemia, hypertension, cardiovascular diseases, and baseline laboratory values (hemoglobin, WBC, neutrophil, ferritin, CRP, ESR). Vascular ultrasound parameters included artery and vein diameters and presence of calcifications. All laboratory variables were recorded using standardized assays performed at our institution. Units and normal reference ranges were as follows: hemoglobin (g/dL, normal: 12–16 g/dL), white blood cell (WBC) count (×10^3^/µL, normal: 4.0–10.0 × 10^3^/µL), neutrophil percentage (%, normal: 40–70%), ferritin (ng/mL, normal: 30–400 ng/mL), C-reactive protein (CRP; mg/L, normal: <5 mg/L), and erythrocyte sedimentation rate (ESR; mm/h, normal: 0–20 mm/h).

The primary outcome was AVF occlusion, defined as the occurrence of vascular access failure necessitating angiographic evaluation and intervention (e.g., percutaneous transluminal angioplasty or thrombectomy) during follow-up. Data were anonymized, and missing values were analyzed to assess patterns. In this study, AVF occlusion was defined as the loss of patency requiring therapeutic intervention, including percutaneous transluminal angioplasty or thrombectomy. Procedural records frequently documented access dysfunction using general terms such as ‘AVF obstruction’ without consistently distinguishing between stenosis and thrombosis; therefore, these conditions were analyzed collectively rather than as separate outcomes.

### 2.3. Missing Data and Imputation

The proportion of missing data for the variables used in model development ranged from 0% to 6.8%. Data were complete for demographic and comorbidity variables, whereas laboratory variables showed low-to-moderate missingness (CRP: 6.8%; ESR: 5.9%; ferritin: 4.7%; neutrophil percentage: 2.9%; WBC: 1.4%; hemoglobin: 1.2%). Missing values in vascular ultrasound parameters ranged from 2.1% to 3.6%. Because missingness was associated with observed clinical characteristics (e.g., comorbidities, baseline inflammatory status) but not with unobserved outcomes, we considered the Missing At Random (MAR) assumption to be appropriate. Therefore, missing data were handled using multiple imputation by chained equations (MICEs), generating five imputed datasets. A sensitivity analysis comparing model performance before and after imputation showed minimal differences (AUC change ≤ 0.01), supporting the robustness of the imputation approach.

### 2.4. Model Development and Evaluation

We evaluated multiple machine learning algorithms for the binary classification of AVF occlusion, including Logistic Regression, Random Forest, XGBoost, CatBoost, and LightGBM. Hyperparameters for all machine learning models were optimized using a grid search coupled with 5-fold stratified cross-validation on the training data. The grid search systematically evaluated predefined parameter combinations, and final hyperparameters were selected based on the highest mean AUC across folds. Hyperparameters were set as follows:

➢ XGBoost: 300 estimators; learning rate: 0.05; max depth: 4; subsample: 0.8; column sampling: 0.8.
➢ Random Forest: 300 trees; max depth: 8.
➢ Logistic Regression: maximum number of iterations: 1000.
➢ LightGBM: 300 estimators; learning rate: 0.05.
➢ CatBoost: 300 iterations; learning rate: 0.05; depth: 6.

Predictive performance was evaluated on the testing dataset using multiple metrics: accuracy, area under the receiver operating characteristic curve (AUC), confusion matrix, precision, recall (sensitivity), specificity, and F1-score. To assess the stability of each model, 5-fold stratified cross-validation was performed on the training data, reporting the mean and standard deviation of the accuracy and AUC. Calibration curves were constructed to compare predicted probabilities of AVF occlusion with observed event rates across risk strata for each model. Receiver operating characteristic (ROC) curves were generated to visualize and compare discriminatory performance.

### 2.5. Statistical Analysis

Baseline characteristics were summarized using means with standard deviations for quantitative variables, and frequency with percentages for qualitative variables. The group comparison employed a *t*-test or chi-square test as appropriate. To enhance interpretability, Shapley Additive Explanations (SHAP) analysis was performed, primarily focusing on the best model due to its superior performance. Mean absolute SHAP values quantify the overall contribution of each predictor to the model’s output. Summary plots depicted both the magnitude and direction of each variable’s effect on predicted risk, with color coding representing high (red) and low (blue) feature values. This approach enabled the identification of key risk factors influencing AVF occlusion. Statistical analyses were conducted using Python (version 3.11). A two-sided *p*-value < 0.05 was considered statistically significant.

## 3. Results

### 3.1. Baseline Characteristics

A total of 1498 patients who underwent AVF creation were included in the study, among whom 381 (25.4%) experienced AVF occlusion during the follow-up period, while 1117 (74.6%) maintained AVF patency. The baseline clinical and laboratory characteristics according to AVF status are summarized in Table 1.

There were no significant differences in age or sex distribution between the occlusion and patency groups. The mean age was 63.5 ± 13.2 and 64.9 ± 13.7 years in the occlusion and patency groups (*p* = 0.071), respectively. The proportion of males was slightly higher in the patency group (58.4%) than in the occlusion group (55.1%), but this difference was not statistically significant (*p* = 0.294).

Significant differences were observed in several clinical parameters. The occlusion group had a lower mean body mass index (BMI) compared to the patency group (22.2 ± 3.1 vs. 22.9 ± 3.6, *p* = 0.000). AVF site distribution differed significantly, with a higher proportion of right-sided AVFs in the occlusion group (28.9% vs. 14.5%, *p* = 0.000). Smoking prevalence was higher in the occlusion group (24.9% vs. 13.1%, *p* = 0.000), as was the prevalence of hyperlipidemia (16.0% vs. 6.0%, *p* = 0.000) and hypertension (85.0% vs. 71.8%, *p* = 0.000).

Vascular measurements revealed that the preoperative draining vein diameter (DVD) was larger in the occlusion group (4.47 ± 1.86 mm) compared to the patency group (3.90 ± 1.83 mm, *p* = 0.000), while differences in inflow artery diameter (IAD) were not statistically significant preoperatively. However, the postoperative increase in IAD was greater in the occlusion group (0.154 ± 0.264 mm vs. 0.072 ± 0.268 mm, *p* = 0.000).

Laboratory values also differed significantly: hemoglobin levels were lower in the occlusion group (9.01 ± 0.91 g/dL vs. 9.43 ± 1.35 g/dL, *p* = 0.000), whereas inflammatory markers such as white blood cell count (WBC), neutrophil percentage, ferritin, C-reactive protein (CRP), and erythrocyte sedimentation rate (ESR) were elevated (all *p*-values < 0.01), indicating a higher inflammatory state in patients experiencing AVF occlusion.

### 3.2. Predictive Model Performance

Five machine learning models—LightGBM, CatBoost, XGBoost, Random Forest, and Logistic Regression—were developed to predict AVF occlusion. Table 2 presents a comprehensive comparison of model performance metrics. LightGBM outperformed other models, achieving the highest area under the ROC curve (AUC) of 0.887, accuracy of 85.8%, and F1-score of 0.68. CatBoost and XGBoost demonstrated comparable performance with AUCs of 0.882 and 0.879, respectively. Random Forest showed slightly lower predictive ability (AUC 0.865), while Logistic Regression exhibited the lowest performance (AUC 0.792). While gradient boosting models demonstrated high specificity (93–95%), their sensitivities ranged from 0.57 to 0.60, indicating moderate ability to detect AVF occlusion cases. Logistic Regression showed lower sensitivity (0.43) and precision (0.62), underscoring the superiority of ensemble machine learning techniques in this context.

### 3.3. Calibration and Discrimination

**Figure 1:**
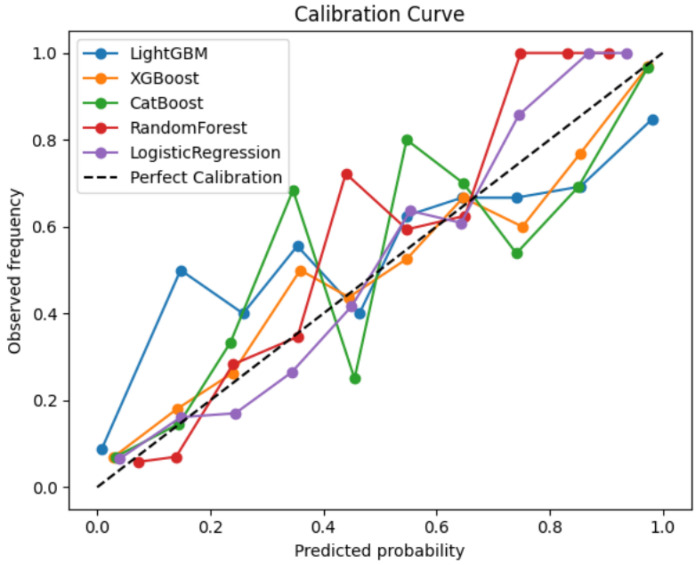
Calibration curves compare the predicted probabilities of AVF occlusion versus observed outcomes for different machine learning models.

**Figure 2:**
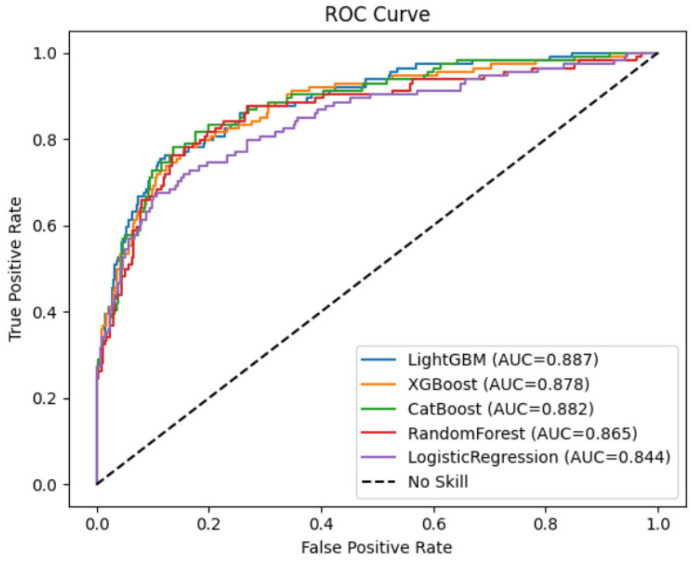
Receiver operating characteristic (ROC) curves illustrating the predictive performance of various machine learning models for AVF occlusion.

Figure 1 shows the calibration curves comparing the predicted probabilities of AVF occlusion with the observed event frequencies across the five machine learning models. Among the models evaluated, LightGBM and CatBoost demonstrated the best calibration, with their curves lying closest to the reference diagonal line representing perfect calibration. This indicates that these models’ predicted probabilities closely matched the actual observed risk of AVF occlusion, suggesting well-calibrated probability estimates suitable for clinical interpretation. Overall, gradient boosting-based algorithms (LightGBM, CatBoost, and XGBoost) provided more reliable and clinically interpretable probability predictions than the conventional Logistic Regression model. In addition, to evaluate discriminative performance, Figure 2 presents the receiver operating characteristic (ROC) curves for the five machine learning models used to predict AVF occlusion. All models demonstrated strong discriminative ability, with areas under the curve (AUC) exceeding 0.84. Among them, LightGBM achieved the highest AUC (0.887), followed by CatBoost (0.882), XGBoost (0.878), Random Forest (0.865), and Logistic Regression (0.844).

The ROC curves of the three gradient boosting-based models (LightGBM, CatBoost, XGBoost) were positioned consistently above the Logistic Regression model, confirming superior classification performance and robustness. The LightGBM model showed particularly high true-positive rates across a wide range of false-positive rates, suggesting more accurate discrimination between patients who experienced AVF occlusion and those who maintained patency.

Together with calibration curve results (Figure 1), these findings indicate that LightGBM and CatBoost not only provided accurate probability estimation but also offered the best balance between sensitivity and specificity for clinical risk prediction.

### 3.4. Feature Importance and Model Interpretability

Feature importance was evaluated using Shapley Additive Explanations (SHAP) analysis applied to the LightGBM model, which demonstrated the best predictive performance.

**Figure 3:**
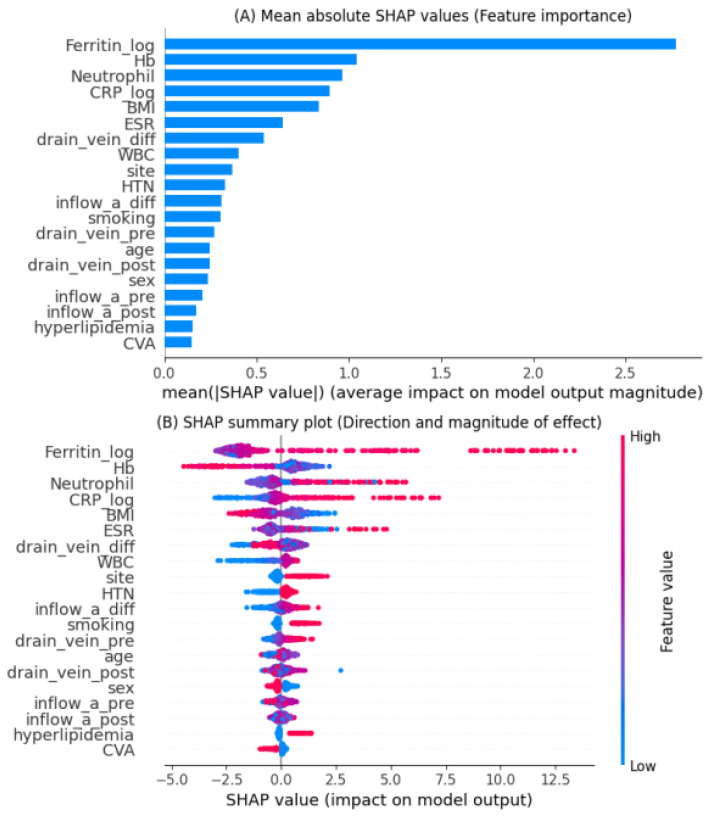
Feature importance analysis based on SHAP values. (**A**) The bar plot shows the mean absolute SHAP value for each feature, representing its overall contribution to the LightGBM model predicting AVF occlusion. (**B**) The summary plot illustrates the direction and magnitude of the effect of each variable. Variables with higher SHAP values indicate stronger influence on the predicted risk; red dots indicate higher feature values, while blue dots indicate lower feature values.

Figure 3 summarizes both the magnitude and direction of the contribution of each feature to AVF occlusion prediction.

Figure 3A shows the mean absolute SHAP values, indicating the overall importance of each variable in the LightGBM model. The most influential predictors were serum ferritin (log-transformed), hemoglobin, neutrophil percentage, and C-reactive protein (CRP, log-transformed). These variables were followed by body mass index (BMI), erythrocyte sedimentation rate (ESR), and the difference in the draining vein diameter before and after surgery, suggesting that both inflammatory markers and vascular characteristics significantly contributed to model predictions.

Figure 3B illustrates the SHAP summary plot, which depicts both the magnitude and direction of each variable’s effect. High ferritin, neutrophil, and CRP levels (represented in red) were associated with an increased predicted risk of AVF occlusion, whereas higher hemoglobin and BMI values (blue) were linked to a lower risk. The results indicate that systemic inflammation and impaired hematologic status are major contributors to AVF dysfunction.

Conversely, vascular features such as postoperative changes in draining vein and inflow artery diameters also influenced risk estimation, reflecting the impact of surgical and anatomical factors.

Overall, SHAP analysis provided an interpretable framework linking biological plausibility with model outputs, enabling the identification of clinically relevant predictors and supporting the potential use of the model for individualized risk stratification.

## 4. Discussion

This study developed and validated machine learning-based predictive models for postoperative arteriovenous fistula (AVF) occlusion using a large cohort of 1498 patients who underwent AVF creation between 2015 and 2025. Among the five models tested, gradient boosting-based algorithms—particularly LightGBM and CatBoost—demonstrated superior predictive performance, achieving the highest accuracy, discrimination, and calibration compared to traditional Logistic Regression and Random Forest models. The results highlight the feasibility and clinical potential of applying explainable artificial intelligence (AI) techniques to predict AVF dysfunction in hemodialysis patients.

Previous studies have primarily employed traditional regression-based approaches to identify risk factors for AVF dysfunction [6,7]. Although these models have provided valuable insights, they were limited by small sample sizes and an inability to account for complex, nonlinear relationships among clinical variables. In contrast, our study incorporated diverse preoperative and perioperative features, including demographic, vascular, and inflammatory parameters, allowing the machine learning models to capture multidimensional interactions. The superior discrimination of LightGBM (AUC: 0.887) and CatBoost (AUC: 0.882) confirms the strength of ensemble learning in handling heterogeneous clinical data.

SHAP analysis revealed that elevated ferritin, neutrophil percentage, and C-reactive protein (CRP) were the most influential predictors of AVF occlusion, whereas higher hemoglobin and body mass index (BMI) were associated with lower risk. These findings are biologically plausible and consistent with prior reports indicating that systemic inflammation and oxidative stress contribute to neointimal hyperplasia and thrombosis in AVFs [6,15]. High ferritin levels may reflect chronic inflammation and iron overload, both of which promote vascular injury and impaired endothelial function. Similarly, CRP and neutrophil activation have been linked to endothelial dysfunction and hypercoagulability, predisposing to vascular access failure. In addition, vascular parameters such as changes in inflow artery and draining vein diameters also contributed meaningfully to the predictive model. These anatomical factors likely reflect surgical technique and vessel adaptability, which are critical determinants of postoperative patency. Therefore, the integration of both biological and anatomical variables underscores the importance of a holistic approach to AVF outcome prediction.

Interestingly, the preoperative draining vein diameter (DVD) was larger in the occlusion group, which contrasts with conventional expectations that smaller veins are associated with poorer AVF outcomes. Several explanations may account for this finding. Patients with long-standing hemodialysis, chronic inflammation, or repeated vascular access manipulation may develop venous dilation that reflects underlying venous hypertension rather than favorable vascular health. In such cases, a larger vein may actually indicate prior stenosis or impaired venous compliance, both of which increase susceptibility to access dysfunction. In addition, operator preference may have led surgeons to select larger but diseased or fibrotic veins in anatomically complex cases, inadvertently increasing the risk of postoperative occlusion. These considerations may help explain why a larger preoperative vein diameter was associated with AVF failure in our cohort.

Ferritin, neutrophil percentage, and CRP emerged as major predictors of AVF occlusion, and their associations are biologically plausible. Elevated ferritin reflects not only iron stores but also chronic inflammation and oxidative stress, both of which promote endothelial injury and impair vascular repair mechanisms. Increased neutrophil levels suggest heightened inflammatory activation, with neutrophil–endothelial interactions accelerating microvascular damage, promoting platelet aggregation, and contributing to a prothrombotic milieu. Similarly, elevated CRP is known to impair endothelial nitric oxide production, enhance smooth muscle proliferation, and stimulate neointimal hyperplasia—all key mechanisms leading to stenosis and thrombosis in AVFs. Collectively, these inflammatory pathways compromise vascular remodeling and increase susceptibility to access dysfunction, supporting their strong predictive contribution in our model.

A major advantage of this study lies in the use of explainable machine learning through SHAP analysis, which allows for transparent interpretation of model predictions. The ability to quantify each feature’s contribution enables clinicians to identify high-risk patients early and implement targeted interventions—such as intensified monitoring, preemptive angioplasty, or aggressive management of inflammation and anemia. Calibration analysis further confirmed that LightGBM and CatBoost produced well-calibrated probability estimates, making them suitable for clinical decision support applications.

Although the gradient boosting models demonstrated strong overall performance with high specificity (0.93–0.95), model sensitivity remained modest (0.57–0.60). This trade-off reflects the clinical balance between minimizing false-positive predictions and ensuring the detection of true occlusion events. In the context of AVF care, high specificity reduces unnecessary imaging or interventions, which is beneficial for patient comfort and healthcare resource utilization. However, moderate sensitivity indicates that a subset of patients at risk for occlusion may not be identified by the model. This limitation highlights the need for threshold optimization or risk-stratified decision frameworks in clinical deployment. Incorporating longitudinal variables such as flow rate trends or repeated inflammatory markers may further enhance sensitivity in future models.

The strengths of this study include the use of a large real-world dataset spanning 10 years, multiple machine learning algorithms for comparative analysis, and explainable AI for model interpretation. However, several limitations should be acknowledged. First, this was a single-center retrospective study, which may restrict the generalizability of our findings to other clinical environments with different patient characteristics, surgical techniques, or postoperative management practices. Second, the model was internally validated but not externally tested; therefore, its performance and robustness in independent, multi-center cohorts remain uncertain. External validation using diverse institutional datasets is essential before the model can be applied broadly in clinical practice. Third, some potentially informative predictors—such as detailed intraoperative factors, postoperative hemodynamic monitoring, and longitudinal vascular access measurements—were unavailable in our dataset, which may have limited the model’s predictive depth. Future work incorporating richer clinical variables and external datasets will be necessary to enhance the model’s accuracy and real-world applicability.

Beyond predictive performance, the model has potential utility in real-world clinical workflows. The risk estimates generated by the LightGBM model could be integrated into electronic medical record (EMR) systems to provide automated, real-time alerts for patients at elevated risk of AVF occlusion. Such risk stratification may help guide preoperative decision-making regarding vein selection or the need for preemptive imaging, and it may also support postoperative management through the identification of patients who would benefit from intensified surveillance, early duplex ultrasound evaluation, or proactive medical optimization of inflammatory status and anemia. In offering interpretable, individualized risk profiles through SHAP analysis, the model may facilitate personalized vascular access planning and more efficient allocation of clinical resources.

Future research should focus on prospective validation and dynamic modeling that incorporates longitudinal variables such as flow rate changes and repeated laboratory measurements. Integration of these models into electronic health record systems could enable real-time risk prediction and individualized clinical decision-making.

## 5. Conclusions

In summary, this study demonstrates that machine learning, particularly gradient boosting algorithms such as LightGBM and CatBoost, can effectively predict AVF occlusion using routinely collected clinical and laboratory data. The incorporation of interpretable AI methods enhances transparency and clinical trust, providing a foundation for precision vascular access management and improved hemodialysis outcomes.

All values were presented as mean ± standard deviation or frequency (percent). *p*-values were obtained using a two-sample *t*-test or chi-square test for quantitative or qualitative variables, respectively.

## Figures and Tables

**Table 1:** Baseline characteristics of patients according to AVF patency and occlusion.

Variable		Patency (*n* = 1117)	Occlusion (*n* = 381)	*p*-Value
Age (years)		64.925 ± 13.722	63.493 ± 13.227	0.071
Sex	Female	465 (41.6)	171 (44.9)	0.294
	Male	652 (58.4)	210 (55.1)	
BMI (kg/m^2^)		22.935 ± 3.617	22.228 ± 3.137	0.000
AVF site	Left	955 (85.5)	271 (71.1)	0.000
	Right	162 (14.5)	110 (28.9)	
Smoking	No	971 (86.9)	286 (75.1)	0.000
	Yes	146 (13.1)	95 (24.9)	
Drinking	No	1029 (93.0)	346 (91.1)	0.247
	Yes	77 (7.0)	34 (8.9)	
Hyperlipidemia	No	1050 (94.0)	320 (84.0)	0.000
	Yes	67 (6.0)	61 (16.0)	
Hypertension	No	315 (28.2)	57 (15.0)	0.000
	Yes	802 (71.8)	324 (85.0)	
COPD	No	1108 (99.2)	381 (100.0)	0.170
	Yes	9 (0.8)	0 (0.0)	
CVD	No	946 (84.7)	330 (86.6)	0.407
	Yes	171 (15.3)	51 (13.4)	
CVA	No	838 (75.0)	322 (84.5)	0.000
	Yes	279 (25.0)	59 (15.5)	
IAD (mm)	Pre-op	4.235 ± 0.957	4.250 ± 0.886	0.788
	Post-op	4.352 ± 0.946	4.417 ± 0.914	0.239
	Difference	0.072 ± 0.268	0.154 ± 0.264	0.000
DVD (mm)	Pre-op	3.903 ± 1.831	4.469 ± 1.862	0.000
	Post-op	4.653 ± 1.829	4.938 ± 1.830	0.009
	Difference	0.679 ± 0.710	0.632 ± 0.558	0.185
Hemoglobin (g/dL; normal: 12–16)		9.427 ± 1.354	9.009 ± 0.912	0.000
WBC (×10^3^/µL; normal: 4.0–10.0)		7.869 ± 3.110	8.400 ± 2.545	0.001
Neutrophil (%; normal: 40–70)		70.996 ± 8.891	75.389 ± 12.874	0.000
Ferritin (ng/mL; normal: 30–400)		439.388 ± 419.586	1075.920 ± 803.381	0.000
CRP (mg/L; normal: <5)		22.804 ± 47.864	50.724 ± 78.508	0.000
ESR (mm/h; normal: 0–20)		35.527 ± 23.850	42.181 ± 35.418	0.001

**Table 2:** Performance of machine learning models for AVF occlusion prediction.

Model	LightGBM	CatBoost	XGBoost	Random Forest	Logistic Regression
Precision	0.790	0.790	0.760	0.730	0.620
Sensitivity	0.600	0.570	0.600	0.540	0.430
Specificity	0.950	0.950	0.930	0.930	0.910
F1-score	0.680	0.660	0.670	0.620	0.510
Accuracy	0.858	0.853	0.849	0.833	0.789
AUC	0.887	0.882	0.879	0.865	0.792

## Data Availability

The data presented in this study are available on request from the corresponding author under ethical and legal restrictions related to patient confidentiality.

## Abbreviations

AVF: arteriovenous fistula; BMI: body mass index; COPD: Chronic Obstructive Pulmonary Disease; CRP: C-reactive protein; CVA: Cerebrovascular Accident; CVD: cardiovascular disease; DVD: draining vein diameter; ESR: erythrocyte sedimentation rate; IAD: inflow artery diameter.
